# Comprehensive RNA-Seq Analysis of Potential Therapeutic Targets of Gan–Dou–Fu–Mu Decoction for Treatment of Wilson Disease Using a Toxic Milk Mouse Model

**DOI:** 10.3389/fphar.2021.622268

**Published:** 2021-04-15

**Authors:** Taohua Wei, Wenjie Hao, Lulu Tang, Huan Wu, Shi Huang, Yue Yang, Nannan Qian, Jie Liu, Wenming Yang, Xianchun Duan

**Affiliations:** ^1^ Department of Neurology, The First Affiliated Hospital of Anhui University of Chinese Medicine, Hefei, China; ^2^ Graduate School, Anhui University of Chinese Medicine, Hefei, China; ^3^ Key Laboratory of Xin'An Medicine, Ministry of Education, Hefei, China; ^4^ Scientific Research and Experiment Center, Anhui University of Chinese Medicine, Hefei, China; ^5^ Institute for Medical Virology, Goethe University Frankfurt Am Main, Frankfurt, Germany; ^6^ Department of Pharmacy, The First Affiliated Hospital of Anhui University of Chinese Medicine, Hefei, China

**Keywords:** Wilson disease (WD), toxic milk mice (TX mice), Gan–Dou–Fu–Mu decoction (GDFMD), RNA-sequencing, traditional Chinese medicine (TCM)

## Abstract

**Background:** Gan–Dou–Fu–Mu decoction (GDFMD) improves liver fibrosis in experimental and clinical studies including those on toxic mouse model of Wilson disease (Model). However, the mechanisms underlying the effect of GDFMD have not been characterized. Herein, we deciphered the potential therapeutic targets of GDFMD using transcriptome analysis.

**Methods:** We constructed a tx-j Wilson disease (WD) mouse model, and assessed the effect of GDFMD on the liver of model mice by hematoxylin and eosin, Masson, and immunohistochemical staining. Subsequently, we identified differentially expressed genes (DEGs) that were upregulated in the Model (Model vs. control) and those that were downregulated upon GDFMD treatment (compared to the Model) using RNA-sequencing (RNA-Seq). Biological functions and signaling pathways in which the DEGs were involved were determined by gene ontology (GO) and Kyoto encyclopedia of genes and genomes (KEGG) pathway analyses. A protein–protein interaction (PPI) network was constructed using the STRING database, and the modules were identified using MCODE plugin with the Cytoscape software. Several genes identified in the RNA-Seq analysis were validated by real-time quantitative PCR.

**Results:** Total of 2124 DEGs were screened through the Model vs. control and Model vs. GDFMD comparisons, and dozens of GO and KEGG pathway terms modulated by GDFMD were identified. Dozens of pathways involved in metabolism (including metabolic processes for organic acids, carboxylic acids, monocarboxylic acids, lipids, fatty acids, cellular lipids, steroids, alcohols, eicosanoids, long-chain fatty acids), immune and inflammatory response (such as complement and coagulation cascades, cytokine–cytokine receptor interaction, inflammatory mediator regulation of TRP channels, antigen processing and presentation, T-cell receptor signaling pathway), liver fibrosis (such as ECM-receptor interactions), and cell death (PI3K-Akt signaling pathway, apoptosis, TGF-beta signaling pathway, etc.) were identified as potential targets of GDFMD in the Model. Some hub genes and four modules were identified in the PPI network. The results of real-time quantitative PCR analysis were consistent with those of RNA-Seq analysis.

**Conclusions:** We performed gene expression profiling of GDFMD-treated WD model mice using RNA-Seq analysis and found the genes, pathways, and processes effected by the treatment. Our study provides a theoretical basis to prevent liver fibrosis resulting from WD using GDFMD.

## Introduction

Wilson disease (WD), also known as hepatolenticular degeneration, is a rare autosomal recessive genetic disease of the nervous system related to disorders of copper metabolism. The clinical manifestations of this disease include liver damage, extrapyramidal symptoms, pigmented corneal rings, and kidney damage. The worldwide incidence rate is about 1 in 30,000 individuals (Bandmann et al., 2015). It is reported that 40–60% of patients with WD have liver disease as the first symptom, and about 50% of patients eventually develop liver cirrhosis (Ferenci et al., 2019). The liver is the central organ where copper is metabolized, and liver fibrosis is the most basic pathological change in the liver caused by disorders of copper metabolism (Bandmann et al., 2015). Liver fibrosis in WD is a pathological process in which the extracellular matrix (ECM) is reversibly deposited in the liver after chronic injury caused by copper ions. It is the main pathological change in almost every WD patient, and is an essential stage in the progression to cirrhosis (European Association for Study of Liver, 2012; Gerosa et al., 2019). Clinically, the main cause of death of patients with WD is liver cirrhosis and complications associated with it. Treatment of such patients with metal chelating agents often needs to be stopped because of severe side effects (Gerosa et al., 2019). This is a clinical problem that needs to be solved urgently and is, therefore, a focus of research globally.

Despite concerns about the need to protect the liver of patients with WD, no major breakthrough has been achieved, and treatment of such patients is still very difficult. It is, therefore, important to find key molecular targets for prevention of liver fibrosis in WD. Both prevention and treatment have great clinical significance. Because the mechanism underlying the pathogenesis of liver fibrosis is complicated, the repair response after liver injury involves the whole body; for this reason, drugs developed for a single target are seldom clinically effective. There are no chemical or biological drugs with prominent curative effects for clinical application. In recent years, research on traditional Chinese medicine (TCM) and its use has revealed therapeutic advantages of these medicines in the prevention and treatment of liver fibrosis (Chen et al., 2016).

In China, Gan–Dou–Fu–Mu decoction (GDFMD), a formula of TCM herbs, has been clinically used to treat WD (Yang et al., 2014). It can improve liver function and liver fibrosis indices in patients with WD exhibiting liver fibrosis. This formula consists of seven commonly used herbs, namely *Reynoutria multiflora* (Thunb.) Moldenke (Zhiheshouwu in Chinese, ZHSW), *Lycium barbarum* L (Gouqi in Chinese, GQ), *Panax notoginseng* (Burkill) F.H.Chen (Sanqi in Chinese, SQ), *Curcuma aromatica* Salisb (Yujin in Chinese, YJ), *Smilax glabra* Roxb (Tufuling in Chinese, TFL), *Bupleurum chinense* DC (Chaihu in Chinese, CH), and *Paeonia lactiflora* Pall (Baishao in Chinese, BS) (Tang et al., 2018). The anti-liver fibrosis effect of GDFMD has been reported to involve regulation of the expression of factors related to the TGF-β1/Smad signaling pathway at gene and protein levels (Yang et al., 2018). However, the specific molecular mechanisms underlying the effects of GDFMD are not completely understood. Therefore, in the present study, we tried to comprehensively elucidate the potential molecular mechanisms underlying the effects of GDFMD on a mouse model of WD using RNA-Seq technology.

## Materials and Methods

### HPLC–MS/MS Analysis of GDFMD

The proportion of the seven herbs in GDFMD was as follows: ZHSW:GQ:SQ:YJ:TFL:CH:BS = 4:20:3:12:12:12:15. All the herbs were obtained from Anqing Huashi Chinese Herbal Medicine Beverage Co., Ltd (Anqing City, China). The steps in the preparation of GDFMD were as follows: First, all the herbal medicines were immersed in water (10-times the weight of medicine for 1 h, and then decocted by boiling for 2 h. After filtration, the residue was decocted with water (8-times the weight of residue) for 1.5 h. The two decoctions were combined and concentrated to 0.2 gmL^−1^ in vacuum at 50°C to obtain the GDFMD suspension. Thereafter, a 1.0 ml aliquot of GDFMD was added to 9.0 ml ethanol, and the mixture was centrifuged at 8000 × *g* for 5 min and filtered through a 0.22 µm membrane. Seven reference substances, namely gallic acid, resveratrol, quercetin, curcumin, paeoniflorin, saikosaponin A, and notoginsenoside (the purity of each compound was determined to be more than 98%), were obtained from Mansite Biotech Co., Ltd (Chengdu, China). The reference substances were weighed and dissolved in 90% ethanol to prepare a mixed reference solution. Two microliter aliquots of sample solutions and the mixed reference solution were used for LC-MS/MS analysis.

An Agilent 1290 HPLC system, coupled with an Agilent 6460 series QqQ/MS with electrospray ionization (ESI) source, was employed as the HPLC-MS/MS system for the quality control of GDFMD. The separation of complex components was performed on a BEH C18 column (100 mm × 2.1 mm, 1.7 µm) maintained at 35°C. Formic acid (0.1%) in water (A) and acetonitrile (B) were used as elution solvents. The gradient elution procedure was as follows: 0–30 min, 5–25% B; 30–40 min, 25–45% B; and 40.01–45 min, 5% B. The flow rate was 0.3 mlmin^−1^. The mass spectrometry was carried by multiple reaction monitoring (MRM) in the ESI-mode. The optimum values of MS parameters were as follows: nebulizing gas pressure, 25 psi; drying gas (N_2_) flow rate, 10 Lmin^−1^; capillary temperature, 350°C. All the data were analyzed using the Mass Hunter workstation software (Agilent Technologies, United States).

### Animal Experiments and Sample Collection

Male Toxic Milk (TX) mice (verified specific-pathogen-free, 20–30 g) were purchased from the Jackson Laboratory (USA). All the mice were genotyped by Sanger sequencing. The mice were randomly divided into the following four groups: normal, model, GDFMD, and penicillamine groups, with 10 mice per group. The mice were anesthetized by intraperitoneal injection of sodium pentobarbital to minimize pain during surgery. The TX model was prepared as described previously (Reed et al., 2018) and the procedure complied with the National Institutes of Health Animal Care and Welfare Guidelines (National Institutes of Health Publication 80–23). The preparation of GDFMD and its administration (6.96 g/kg, once a day) to animals was done as previously described by us (Tang et al., 2019). Four weeks after the interventions, the mice were sacrificed and their liver was excised and promptly placed in liquid nitrogen for cryopreservation. The experimental protocol was authorized by the Animal Experiment Ethics Committee of the Anhui University of Chinese Medicine (2018AH-08).

### Histopathological Examination

The liver was harvested at day 28 after GDFMD administration and fixed with 4% paraformaldehyde. Paraffin-embedded sections were stained with hematoxylin and eosin. Furthermore, we performed Masson staining of the sections and immunohistochemical staining for Col1 and α-SMA.

### RNA Extraction, cDNA Library Preparation, Next-Generation Sequencing

A total RNA isolation kit (TR205–200, Tianmo, CN) was used for extracting the total RNA from liver samples, according to the instructions provided by the manufacturer. The quantity and quality of RNA samples were determined using a NanoDrop one (Thermo Fisher Scientific, United States) and Agilent 2100 Bioanalyzer (Agilent Technologies, United States). The RNA library was prepared using the SureSelect chain-specific RNA library preparation kit (Agilent Technologies, United States). Thereafter, Qubit 3.0 Fluorometer and Agilent 2100 Bioanalyzer were used to quantify the purified libraries. The generation of clusters was performed using cBot, and the sequencing was achieved with Illumina NovaSeq 6000 (Illumina, United States). These procedures were outsourced to Origin-Biotech Inc (Ao-Ji Biotech, Shanghai, China).

### Analysis of Gene Profile Related to the Regulation of WD by GDFMD

FastQC was used for quality control of experimental samples to ensure the quality of RNA-Seq reads (version. 0.11.3). Fastp was used to trim the discovered Illumina TruSeq adaptor sequences, bad quality reads, and ribosomal RNA reads (Chen et al., 2018). The mapping of trimmed reads against the *Mus musculus* reference genome (mm10) was carried out using Hisat (Kim et al., 2015; Pertea et al., 2016). Thereafter, the quantification of each gene was done with read counts from the trimmed reads using the Stringtie (version: 1.3.0) software (Pertea et al., 2015; Pertea et al., 2016). Trimmed means of M values were normalized with gene counts (Robinson et al., 2010), and a Perl script was used to estimate the fragments per kilobase of transcript per million mapped reads (FPKM) (Mortazavi et al., 2008). To visualize the differences in gene expression in the liver tissue from control, tx-j, and GDFMD mice, we performed principal components analysis (PCA) using RNA-Seq expression data. EdgeR was used to analyze and determine the differentially expressed genes (DEGs) (Robinson et al., 2010; Nikolayeva and Robinson, 2014), and significance was determined by setting the following criteria: *p* value <0.05 and |log_2_ (fold-change)| > 1 (Benjamini et al., 2001). The upregulated (up-DEGs; the intersection of Model and control or GDFMD, respectively) and downregulated (down-DEGs; the intersection of Model and control and GDFMD, respectively) genes were filtered out using Venny.

### Functional Classification of Genes Involved in the Regulation of WD by GDFMD

To better understand the functions of DEGs, the R software package was used to analyze the signaling pathways enriched in Gene Ontology (GO) (Ashburner et al., 2000) and the Kyoto Encyclopedia of Genes and Genomes (KEGG) (Kanehisa and Goto, 2000). We used clusterProfiler to analyze the GO terms and KEGG pathways, and top 30 GO terms and pathways are listed (Yu et al., 2012).

### Construction of PPI Network and Module Analysis

STRING, a database of information on protein interactions including the data on predicted and experimentally determined interactions (Szklarczyk et al., 2017), was adopted for determining the interactions of proteins encoded by genes involved in the regulation of WD by GDFMD. Based on interactions with combined scores ≥0.7, PPIs for the DEGs were generated using the STRING tool. Subsequently, visualization of the PPI network was done with Cytoscape (Shannon et al., 2003), and disconnnected nodes have been hidden. Using default conditions for the functional enrichment analysis module, the PPI network was used for screening the module based on the MCOD plugin in Cytoscape (Bader and Hogue, 2003). The clusterProfiler was used to analyze the DEGs enriched in GO and KEGG in the four groups (Yu et al., 2012).

### Validation of Genes Involved in the Regulation of WD by GDFMD Using qRT-PCR

For verifying the reproducibility and reliability of the results of RNA-Seq, qRT-PCR was performed, using *GAPDH* as an internal control. Six GDFMD-regulated genes, also known to participate in liver injury, namely *Timp1*, *Fbn1*, *Gas6*, *Alb*, *Apob*, and *Apoa2*, were selected for qRT-PCR analysis. The expression levels in the liver samples from the control, model, and GDFMD groups were analyzed. The relative expression of mRNAs was determined using the 2^−∆∆CT^ method (Livak and Schmittgen, 2001).

### Statistical Analysis

Raw data are expressed as means ± standard deviation (SD). Statistical analysis of the data was performed using the GraphPad Prism (GraphPad Software, United States). A *p*-value less than 0.05 indicated statistical significance.

## Results

### Identification and Determination of the Main Chemical Components of GDFMD

To ensure the quality and stability of the GDFMD, HPLC-MS/MS was used to detect and identify the chemical components of GDFMD. Seven known components, namely gallic acid (tR ≈ 0.82 min, MW = 169), paeoniflorin (tR ≈ 8.43 min, MW = 479.2), resveratrol (tR ≈ 16.15 min, MW = 226.9), quercetin (tR ≈ 21.92 min, MW = 301), notoginsenoside (tR ≈ 23.10 min, MW = 931.7), saikosaponin A (tR ≈ 37.47 min, MW = 779), and curcumin (tR ≈ 39.78 min, MW = 367.1) were identified by comparing the retention times with those of the reference standards. The MS parameters and the content of these seven components in GDFMD are shown in Table 1. The MRM diagrams of the seven components in the mixed reference solution and GDFMD are shown in Supplementary Figure S1.

**TABLE 1:** Multiple reaction monitoring (MRM) conditions and the contents of seven components in GDFMD.

No	t_R_ (min)	Name	Precursor ion (m/z)	Product ion (m/z)	Dwell time (ms)	Frag. (V)	C.E. (V)	Content (mg/kg)	Source
1	0.82	Gallic acid	169.0	124.9	30	80	18	170	BS
2	8.43	Paeoniflorin	479.2	121.0	30	135	35	200	BS
3	16.15	Resveratrol	226.9	184.7	30	127	24	1.4	ZHSW
4	21.92	Quercetin	301.0	179.0	30	132	22	1.6	TFL
5	23.10	Notoginsenoside	931.7	799.5	30	260	45	106	SQ
6	37.47	Saikosaponin A	779.0	617.0	30	230	50	84	CH
7	39.78	Curcumin	367.1	133.6	30	100	45	0.03	YJ

### Effects of GDFMD on Mouse Model of WD

**FIGURE 1:**
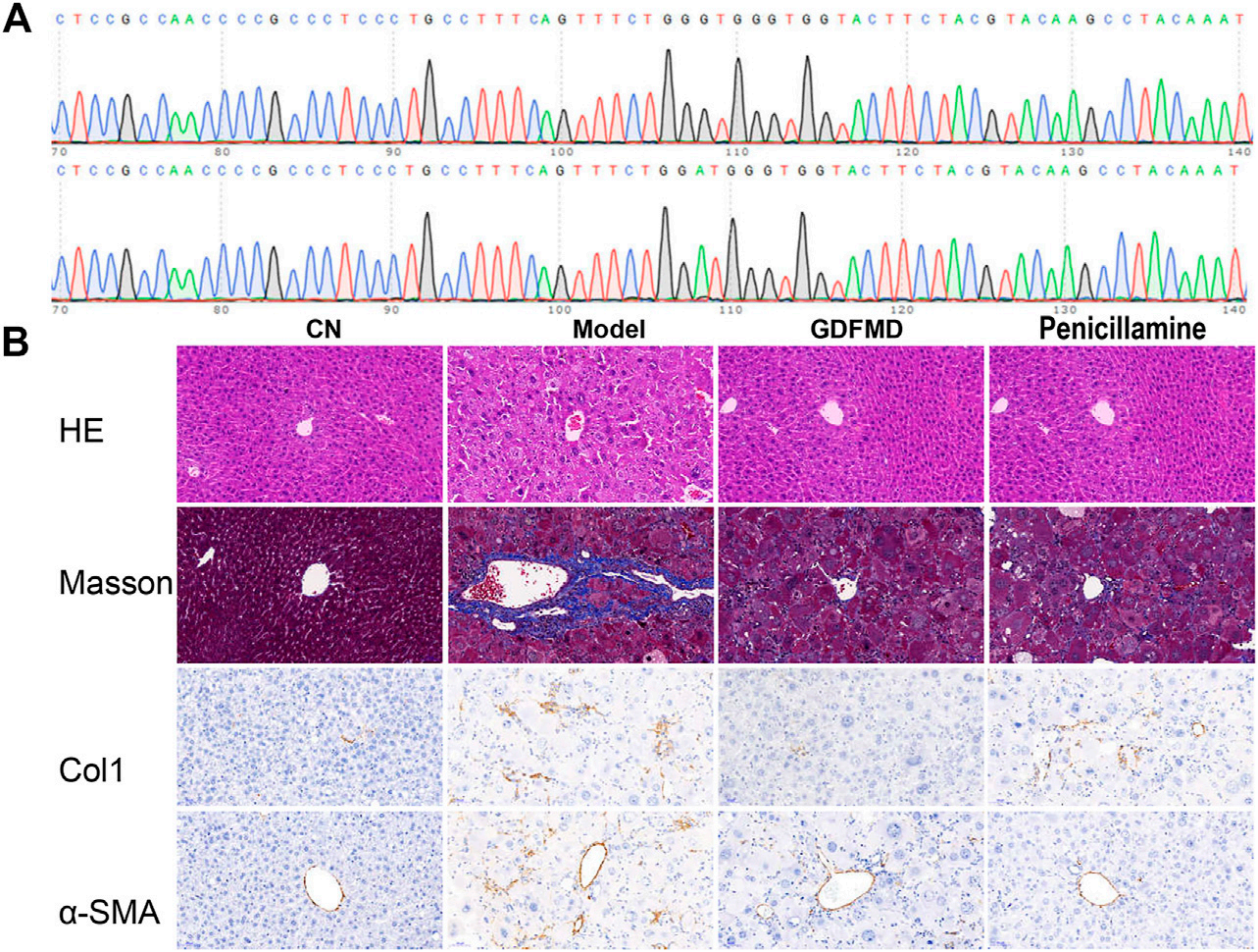
GDFMD reversed DEGs with Model **(A)** The Venn diagram of GDFMD-revised gene with WD. down-DEGs (Model vs. CN and GDFMD vs. Model, down- and up-regulated, respectively) and up-DEGs (Model vs. CN and GDFMD vs. Model, up- and down-regulated, respectively) **(B)** Heatmap of down-DEGs and up-DEGS.

First, all the mice were genotyped. The mice with diploid mutation were tx-j mice (Model group), whereas the diploid wild type mice were used as control mice (control group) (Figure 1A). To observe the protective effects of GDFMD and penicillamine against WD, TX mice were treated with GDFMD and penicillamine, administered intragastrically. The results indicated that WD induced liver injury in the mouse model. The results of HE staining (Figure 1B) indicate that the liver was healthy in the control group, whereas diffuse lesions in the liver were observed in the WD model. A larger number of hepatic parenchyma cells were observed to be necrotic in the tx-j group compared with that in the control. GDFMD and penicillamine could effectively improve these lesions.

**FIGURE 2:**
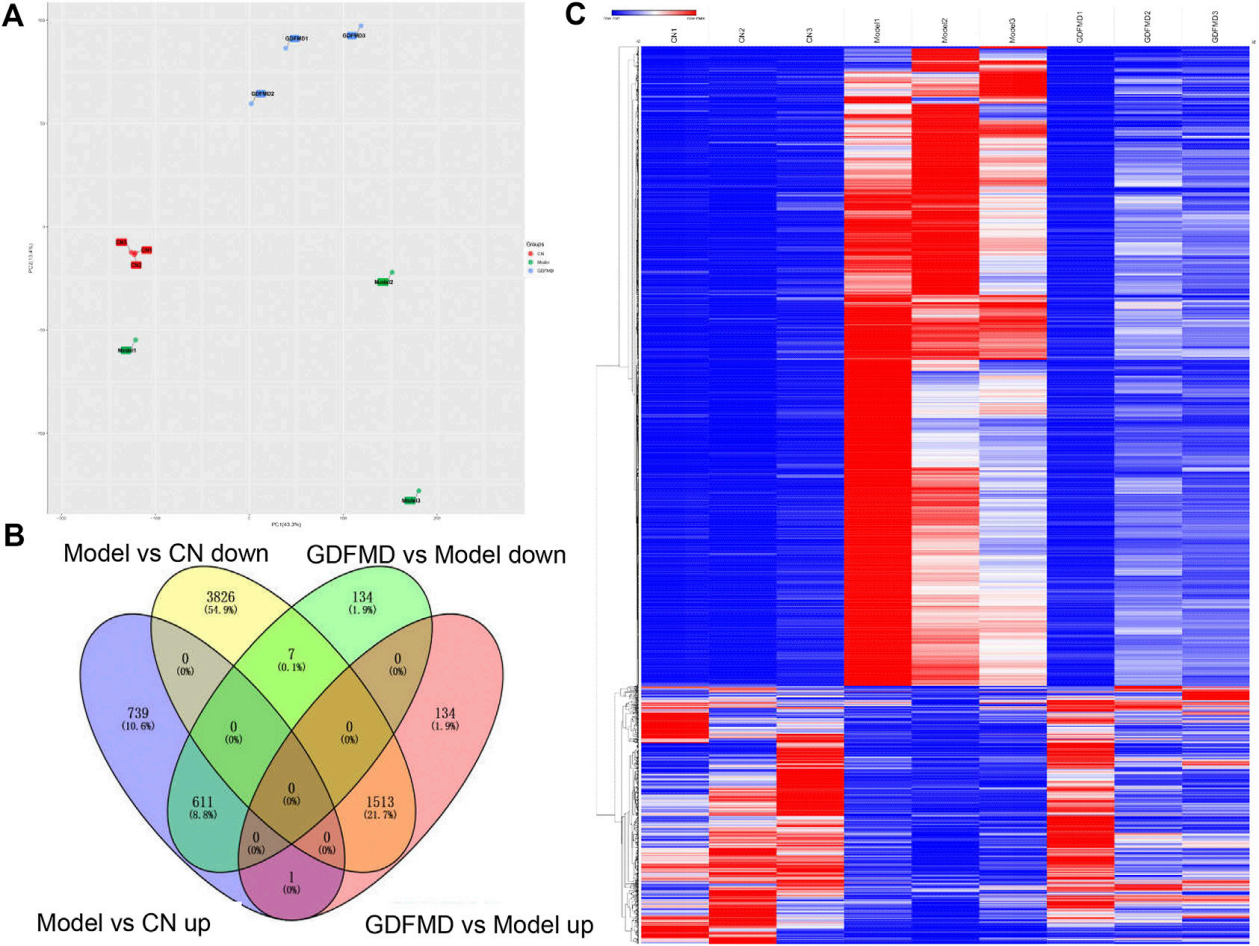
Bubbleplots of the GO enrichment analysis results using ClusterProfiler for down-DEGs **(A)** and down-DEGs **(B)** resulting for GDFMD-revised with WD.

Collagen was stained blue in liver sections with Masson staining as well as with IHC staining for Col1a. The TX mice showed obvious collagen accumulation compared with those in the control group (Figure 2B), and administration of GDFMD could reverse this. α-SMA is a marker of hepatic stellate cell activation, which has been widely used in the study of liver diseases. We, therefore, performed IHC staining for α-SMA. As shown in Figure 1B, the expression of α-SMA was upregulated in the tx-j model group compared with that in the control, and was downregulated in the penicillamine and GDFMD groups. This evidence proves that GDFMD can alleviate the death of liver cells, inflammatory cell infiltration, and liver fibrosis, just like penicillamine, in WD mice.

### Identification of DEGs

To study the mechanism of action of GDFMD in the mice model, we performed RNA-Seq analysis and compared the transcriptomic profiles of the control, Model, and GDFMD groups. PCA showed that control, tx-j, and GDFMD sample groups could segregate into different areas of the 2D plots (Figure 2A). As shown in Figure 2B, 611 down-DEGs that were common among the 1351 downregulated mRNAs in the Model vs. control comparison and 752 downregulated mRNAs in the Model vs. GDFMD comparison were filtered. Similarly, 1513 up-DEGs were identified to be common among the 5346 upregulated mRNAs in the Model vs. control comparison and 1648 upregulated mRNAs in the Model vs. GDFMD comparison (Figure 2B). At last, A total of 2124 DEGs were detected in the Model vs. control and Model vs. GDFMD comparisons, those were GDFMD revised the dyregulated genes with WD. The heatmap of the DEGs expression was shown in Figure 2C.

### GO Analysis of DEGs Altered by GDFMD

**FIGURE 3:**
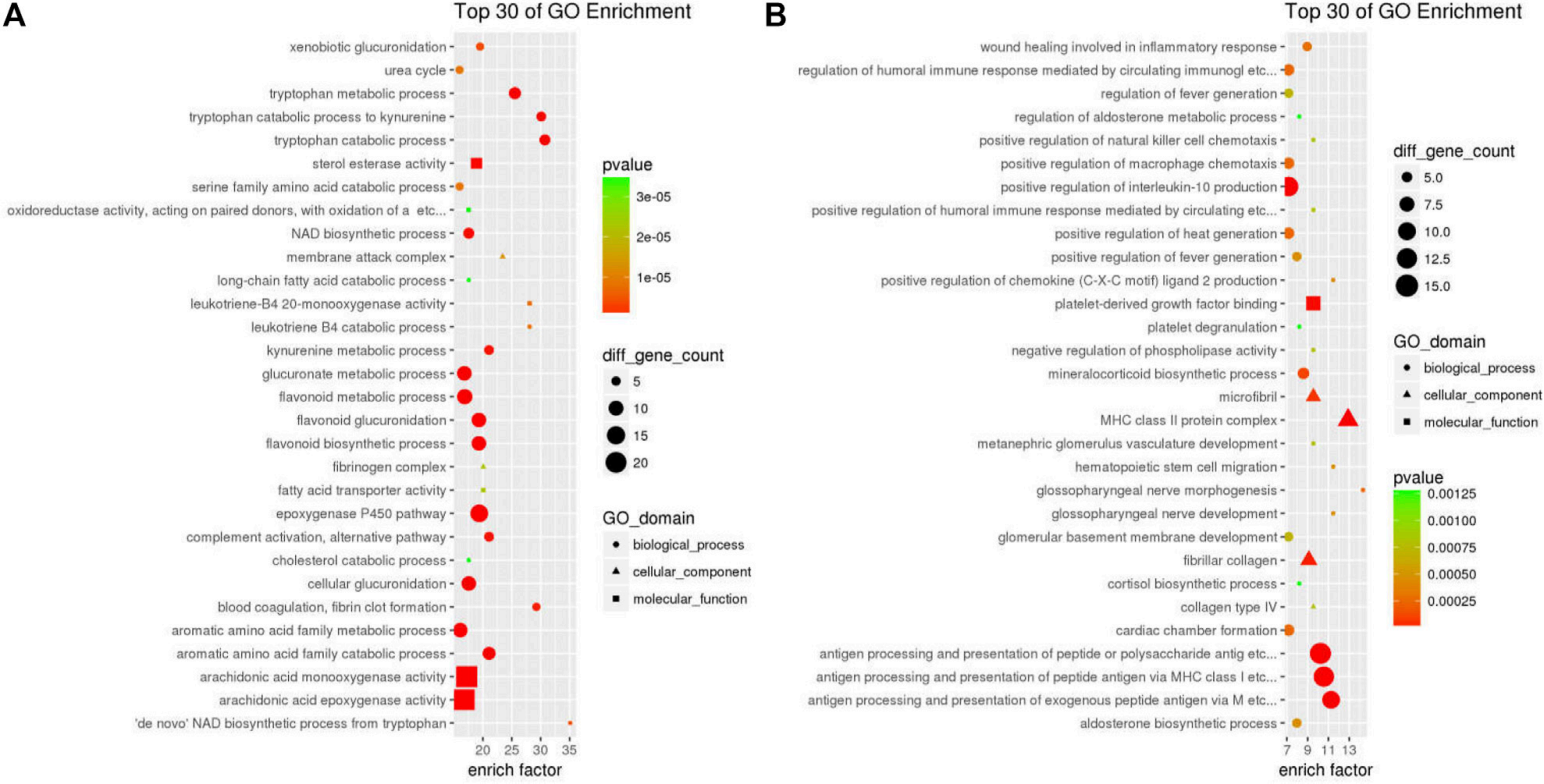
Bubbleplots of the KEGG pathway enrichment analysis results using ClusterProfiler for down-DEGs (**(A)** and down-DEGs **(B)** resulting for GDFMD-revised with WD.

To further identify the 611 down-DEGs and 1513 up-DEGs related to GDFMD treatment in the Model group, GO enrichment analysis was performed using the clusterProfiler. The results of enrichment analysis showed that the 611 down-DEGs were significantly (*p* < 0.05) enriched in 1553 GO terms, 511 of which were related to biological processes (BP), 43 were related to cellular components (CC), and 152 were related to molecular functions (MF). The top five enriched BP terms were organic acid metabolic process (GO: 0006082), carboxylic acid metabolic process (GO: 0019752), monocarboxylic acid metabolic process (GO:0032787), lipid metabolic process (GO:0006629), and oxidation–reduction process (GO:0055114). Blood microparticle (GO: 0072562), extracellular space (GO: 0005615), endoplasmic reticulum (GO: 0005783), mitochondrion (GO: 0005739), and extracellular region (GO: 0005576) were the top five enriched CC terms. The top five enriched MF terms were oxidoreductase activity (GO: 0016491), catalytic activity (GO: 0003824), oxidoreductase activity, acting on paired donors, with incorporation or reduction of molecular oxygen (GO: 0016705), monooxygenase activity (GO: 0004497), and tetrapyrrole binding (GO: 0046906). The top 30 GO terms related to down-DEGs, according to enrichment factor, are listed in Figure 3A.

The 1513 up-DEGs were significantly (*p* < 0.05) enriched in 1631 GO-terms, including 1375 BP, 124 CC, and 132 MF terms. The top five enriched BP terms were cell adhesion (GO:0007155), regulation of cell migration (GO:0030334), cell-cell adhesion (GO:0098609), cellular component movement (GO:0006928), and locomotion (GO:0040011). Moreover, we found that many of the significantly enriched terms were related to collagen, fibril, and fibroblast; these included negative regulation of collagen (GO:0005581, *p* = 4.293e−10), fibrillar collagen (GO:0005583, *p* = 2.577e−05), cellular response to fibroblast growth factor stimulus (GO:0044344, *p* = 7.215e−04), and collagen type IV (GO:0005587, *p* = 7.818e−04). Cell surface (GO:0009986), extracellular matrix (GO:0031012), proteinaceous extracellular matrix (GO:0005578), extracellular region (GO:0005576), and cell periphery (GO:0071944) were the top five enriched CC terms. The enriched MF terms were calcium ion binding (GO:0005509), glycosaminoglycan binding (GO:0005539), heparin binding (GO:0008201), growth factor binding (GO:0019838), and extracellular matrix structural constituent (GO:0005201). The top 30 GO terms related to up-DEGs, according to the enrichment factor, are shown in Figure 3B.

These results suggest that GDFMD may regulate the biology process of metabolic process (eg, metabolic process of organic cyclic compound, carboxylic acid, monocarboxylic acid, lipid, etc), response to stimulus (eg, response to stress, organic substance, external stimulus, hormone, lipid, organic cyclic compound, wounding, cytokine, growth factor, etc), immune and inflammatory response, etc. GDFMD can affect almost all cell components, including intracellular, membrane-bounded organelle, membrane, cytoplasm, cell periphery, vesicle, extracellular vesicle, nucleus, cytoskeleton, etc.

### KEGG Pathway Analysis of DEGs Altered by GDFMD

**FIGURE 4:**
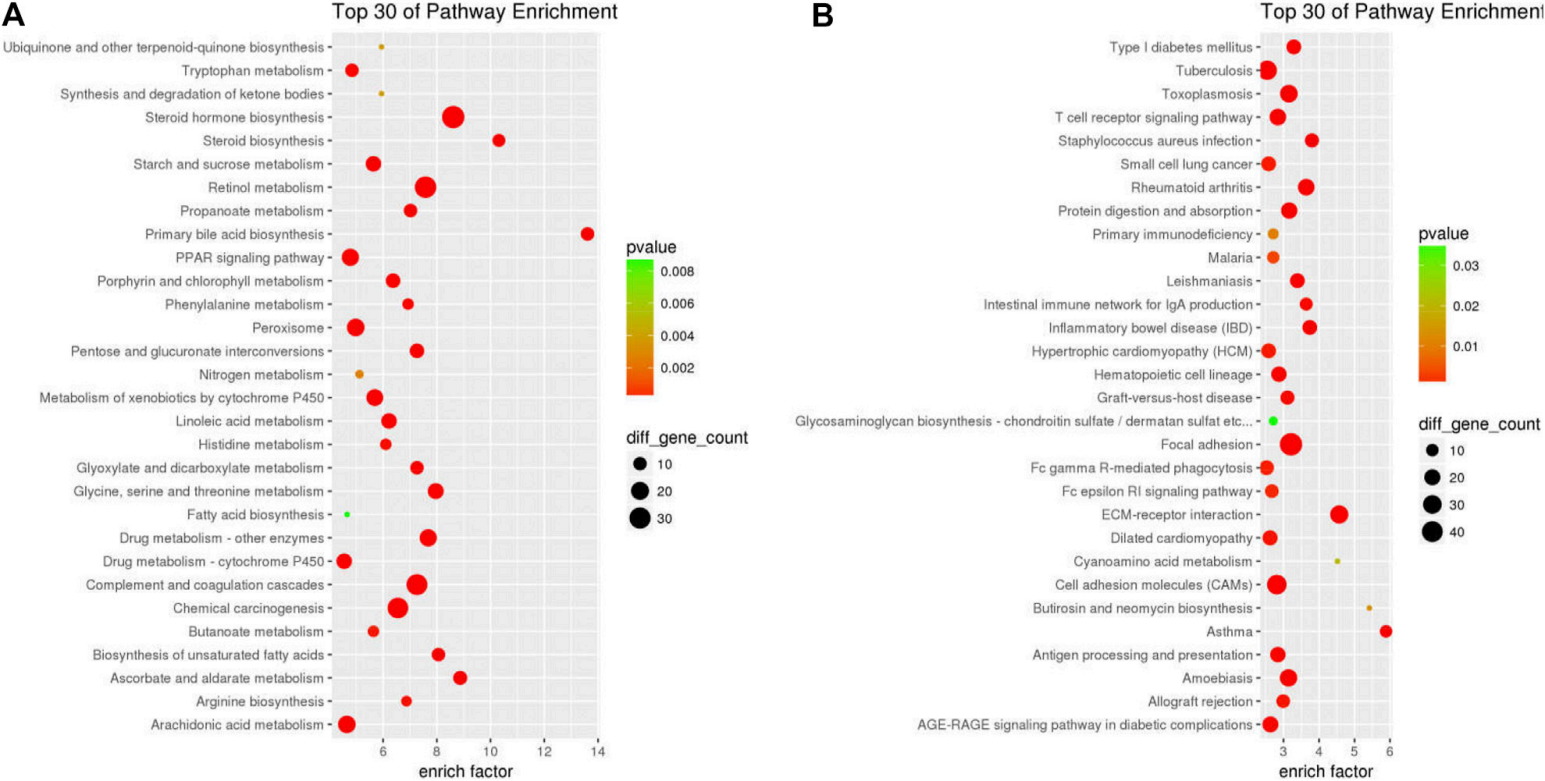
PPI network of the DEGs resulting from GDFMD treatment of WD. Node sizes correlate with node degree; The higher expression genes in WD group that compared with CN and GDFMD group were performed pink nodes, The lower expression genes in WD group that compared with CN and GDFMD group were performed green nodes; PPI, protein-rotein interaction; DEGs, differentially expressed genes.

More information pertaining to the function of genes and interactions among them can be obtained through pathway enrichment analysis. ClusterProfiler was used for KEGG pathway analysis of 611 down-DEGs and 1513 up-DEGs, which were related to the GDFMD intervention in the Model group. The 611 down-DEGs were enriched in 230 pathway terms, including 65 significant terms with *p* < 0.05. In particular, the top10 enriched pathways were metabolic pathways (mmu01100), steroid hormone biosynthesis (mmu00140), retinol metabolism (mmu00830), complement and coagulation cascades (mmu04610), chemical carcinogenesis (mmu05204), drug metabolism - other enzymes (mmu00983), glycine, serine, and threonine metabolism (mmu00260), primary bile acid biosynthesis (mmu00120), metabolism of xenobiotics by cytochrome P450 (mmu00980), and peroxisome (mmu04146). The top 30 KEGG pathway terms related to down-DEGs, according to the FDR-value, are shown in Figure 4A.

The 1513 up-DEGs were enriched in 251 pathway terms including 73 terms with *p*-value < 0.05. The top10 enriched pathways were focal adhesion (mmu04510), ECM-receptor interaction (mmu04512), cell adhesion molecules (CAMs) (mmu04514), rheumatoid arthritis (mmu05323), cytokine-cytokine receptor interaction (mmu04060), toxoplasmosis (mmu05145), amebiasis (mmu05146), PI3K-Akt signaling pathway (mmu04151), tuberculosis (mmu05152), and asthma (mmu05310). The top 30 KEGG pathway terms related to up-DEGs, according to the FDR-value, are shown in Figure 4B.

Thus, dozens of pathways, especially metabolic regulation, immune regulation, extracellular matrix, cell death, and other important pathways, can be affected by GDFMD.

### PPI Network Related to DEGs Altered by GDFMD

**FIGURE 5:**
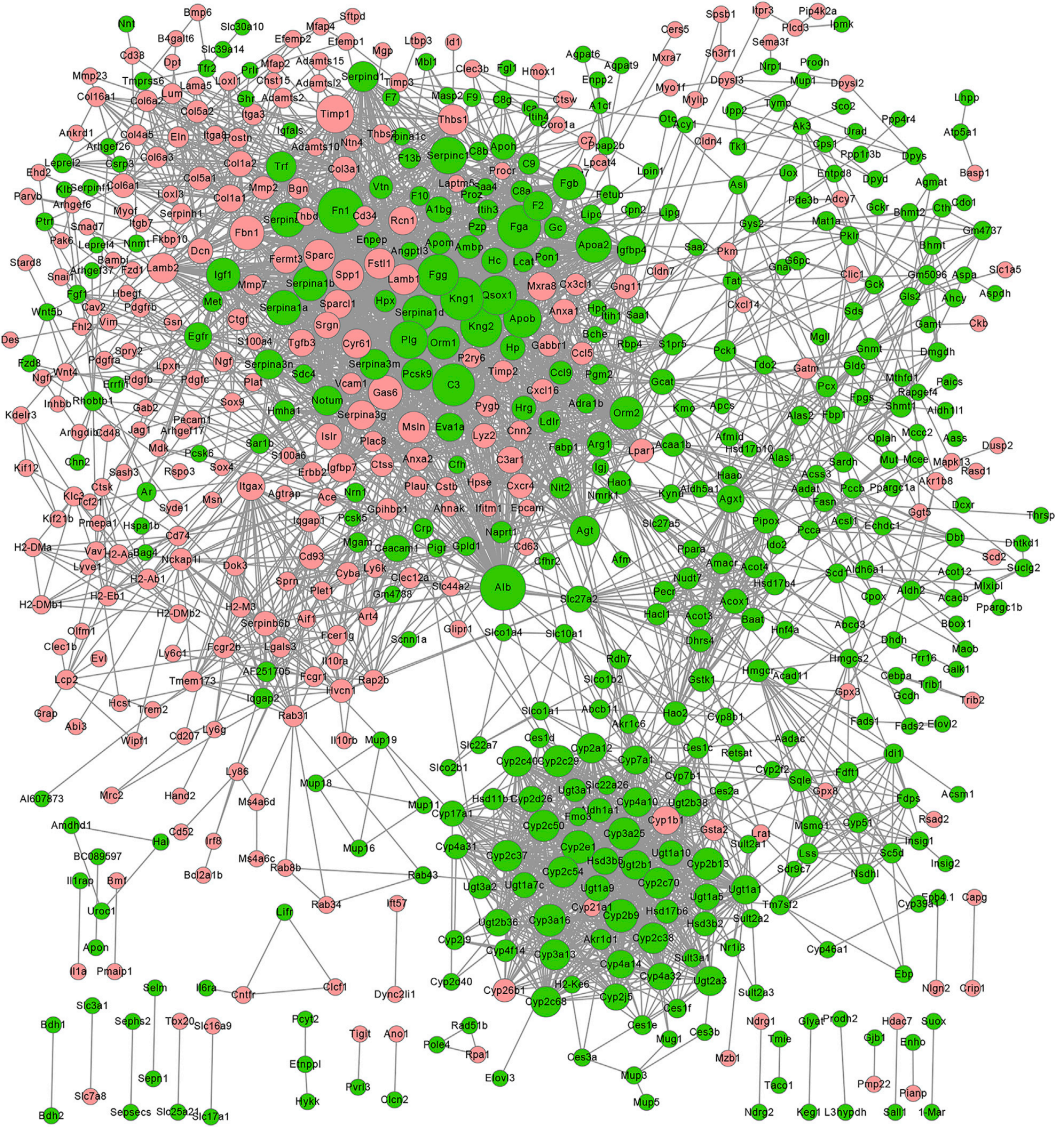
Four key modules network **(A–D)** of PPI network for DEGs reversed by GDFMD with WD. Pink nodes denote up-regulated genes; PPI, protein-protein interaction; DEGs, differentially expressed genes.

We constructed the PPI network related to DEGs altered by GDFMD, according to the data in the STRING database. The PPI network, consisting of 675 DEGs and 1,755 pairs of interactions, is shown in Figure 5. The connectivity degrees were high for dozens of gene nodes, such as *Alb* (albumin, degree = 216), *Plg* (plasminogen, degree = 155), *Fn1* (fibronectin 1, degree = 148), *Fga* (fibrinogen alpha chain, degree = 146), *Apob* (apolipoprotein B, degree = 131), *Agxt* (alanine-glyoxylate and serine-pyruvate aminotransferase, degree = 130), *Kng1* (kininogen 1, degree = 128), *F2* (coagulation factor II, thrombin, degree = 127), *Fgg* (fibrinogen gamma chain, degree = 127), and *Serpinc1* (serpin family C member 1, degree = 115). The details of the top 10 down- and up-regulated genes upon GDMFD administration are provided in Table 2.

**TABLE 2:** Top10 degrees of up- and down-regulated genes in WD.

gene_id	gene_name	Model vs. CN		Model vs. GDFMD		Degree
		log2FC	*P* value	log2FC	*P* value	
ENSMUSG00000029368	Alb	−2.8995	1.65E−15	−2.340268016	8.22E−05	216
ENSMUSG00000059481	Plg	−2.32442	4.27E−11	−1.773976395	0.000238438	155
ENSMUSG00000026193	Fn1	−1.42758	0.00022	−1.357898071	0.002903195	148
ENSMUSG00000028001	Fga	−1.85894	3.18E−06	−1.308745217	0.009843242	146
ENSMUSG00000020609	Apob	−1.06738	0.004563	−1.085623439	0.0056967	131
ENSMUSG00000026272	Agxt	−2.08646	4.75E−11	−1.052025369	0.003392567	130
ENSMUSG00000022875	Kng1	−2.00375	2.39E−09	−1.480563807	0.000780989	128
ENSMUSG00000027249	F2	−1.81617	1.24E−08	−1.678654285	0.000129363	127
ENSMUSG00000033860	Fgg	−2.08137	3.90E−08	−1.297170969	0.006287006	127
ENSMUSG00000026715	Serpinc1	−1.30949	3.87E−05	−1.186936695	0.002791156	115
ENSMUSG00000001131	Timp1	9.44696	9.37E−22	1.948772907	0.017978293	102
ENSMUSG00000029304	Spp1	4.232861	3.12E−30	1.330100157	0.045784578	80
ENSMUSG00000040152	Thbs1	4.883876	7.55E−19	1.778997053	0.00358864	76
ENSMUSG00000027204	Fbn1	3.890804	3.90E−18	1.555481848	0.002069917	73
ENSMUSG00000018593	Sparc	2.664284	2.15E−09	1.453464919	0.001194916	72
ENSMUSG00000001506	Col1a1	6.425957	1.59E−46	2.112437372	0.008277757	65
ENSMUSG00000030789	Itgax	5.103869	3.72E−19	1.382309423	0.024848423	65
ENSMUSG00000035042	Ccl5	3.616753	2.80E−07	1.637814589	0.003349463	62
ENSMUSG00000020717	Pecam1	1.355011	0.001301	1.073938652	0.014971096	61
ENSMUSG00000045382	Cxcr4	2.984457	6.91E−09	1.611341172	0.001304974	60

**FIGURE 6:**
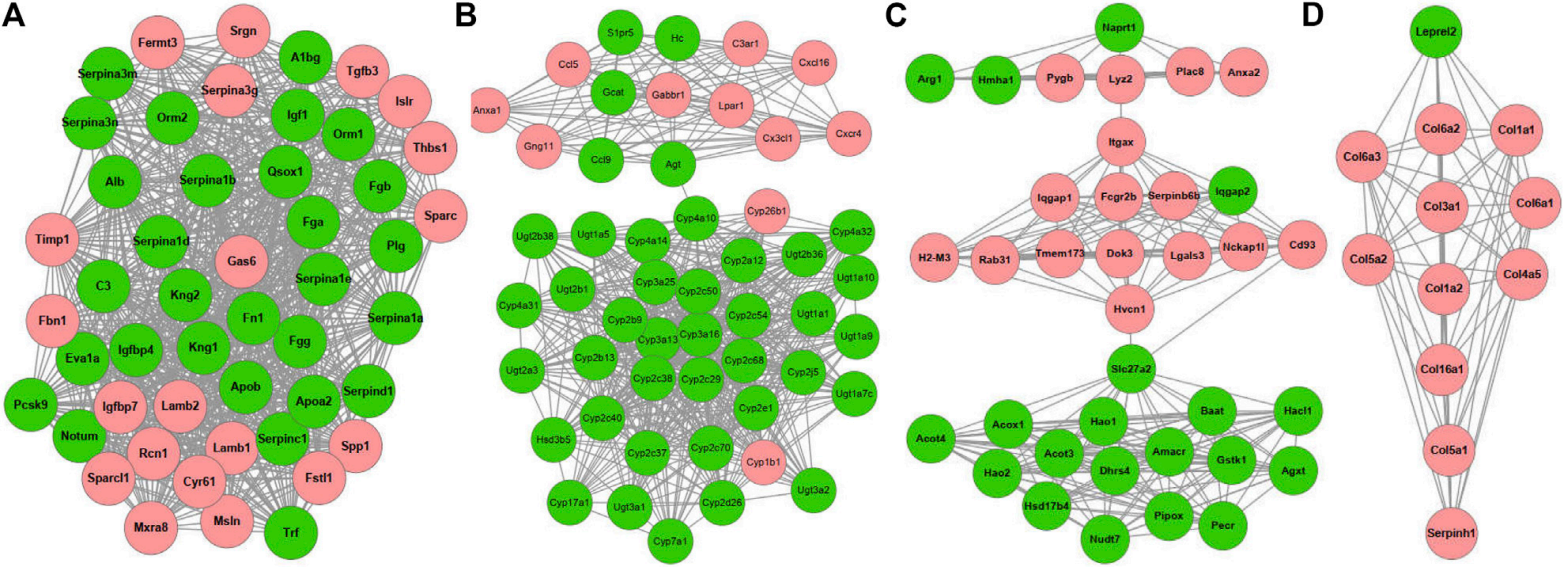
Verification of DEGs by qRT-PCR. Expression of six genes in liver tissues was detected by qRT-PCR, and shown by the expression fold changes for Model and GDFMD vs. CN. Actb was used as the internal control.

Using the MCODE plugin with default criteria, we obtained 39 modules, and these modules are presented based on MCODE scores in descending order. Following the MCODE scores, the top4 modules were designated as modules 1 to 4. These four modules were selected for visualization of the module network (Figure 6). Specifically, module 1 consists of 49 genes and 880 interaction pairs, module 2 consists of 51 genes and 598 interaction pairs, module 3 consists of 36 genes and 222 interaction pairs, and the module 4 consists of 12 genes and 65 interaction pairs.

### qRT-PCR Verification of the DEGs

**FIGURE 7:**
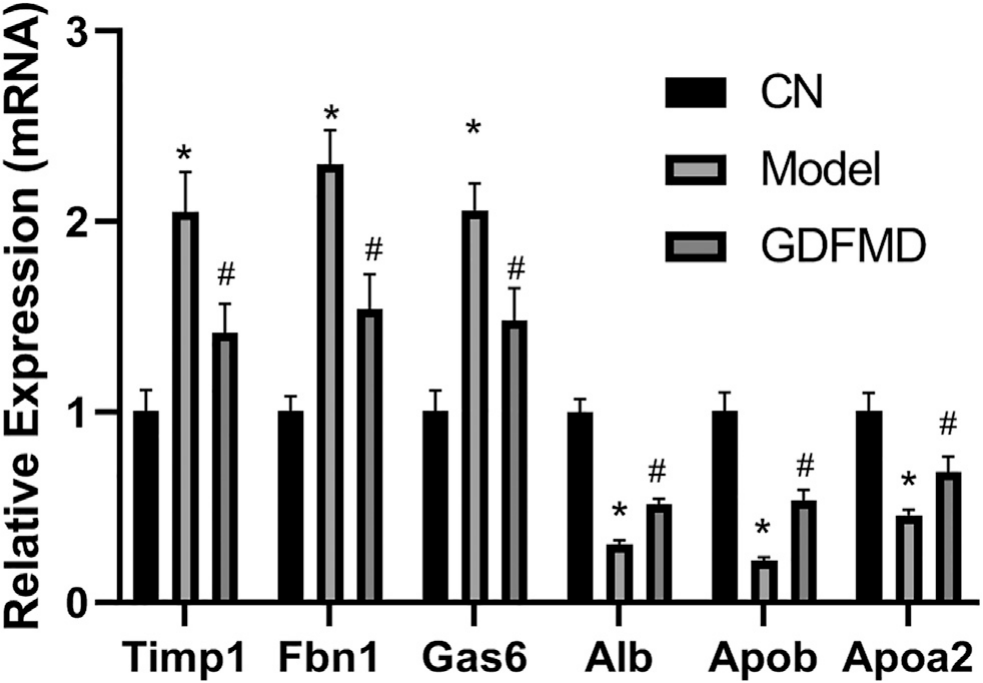
Significantly enriched KEGG pathways in PPAR signaling pathway. Up-DEGs are marked in red. The pictures were drawn by KEGG Mapper (www.kegg.jp/kegg/tool/map_pathway2.html).

Compared with the CN group, the results of qPCR demonstrated that *Timp1*, *Fbn1*, and *Gas6* were overexpressed in the Model group (Figure 7). In contrast, the expression levels of *Alb*, *Apob*, and *Apoa2* were low in the Model group as assessed by the two methods (Figure 7). GDFMD reversed the expression of these DEGs to different extent in the Model group (Figure 7). Thus, the results of RNA-Seq and real-time PCR analyses were consistent, indicating the reliability of the RNA-Seq results. The primers used for qRT-PCR analysis are listed in Table 3.

**TABLE 3:** Primer sequences.

Genes	Primers (5′–3′)		Product length
Timp1	F	CGA​GAC​CAC​CTT​ATA​CCA​GCG	108
	R	ATG​ACT​GGG​GTG​TAG​GCG​TA	
Fbn1	F	TGT​GGG​GAT​GGA​TTC​TGC​TC	165
	R	AGT​GCC​GAT​GTA​CCC​TTT​CTG	
Gas6	F	CCG​CGC​CTA​CCA​AGT​CTT​C	110
	R	CGG​GGT​CGT​TCT​CGA​ACA​C	
Alb	F	CAA​GAG​TGA​GAT​CGC​CCA​TCG	131
	R	TTA​CTT​CCT​GCA​CTA​ATT​TGG​CA	
Apob	F	GCT​CAA​CTC​AGG​TTA​CCG​TGA	190
	R	AGG​GTG​TAC​TGG​CAA​GTT​TGG	
Apoa2	F	GCA​GAC​GGA​CCG​GAT​ATG​C	142
	R	GCT​GCT​CGT​GTG​TCT​TCT​CA	

## Discussion

Several studies have shown that GDFMD treatment can prevent WD in model mice and has clinical application (Yang et al., 2014; Tang et al., 2018; Yang et al., 2018; Tang et al., 2019). In the present study also, we found that GDFMD treatment attenuated the liver injury caused by WD. However, the molecular mechanisms are unclear and require further studies. In the present study, the results of RNA-Seq analysis show that the GDFMD decoction could improve the phenotypic characteristics of WD and alter the expression of several genes. Furthermore, using GO, KEGG pathway, and PPI network analyses, we noticed that most of the genes altered by GDFMD were related to metabolism (including metabolic processes for organic acids, carboxylic acids, monocarboxylic acids, lipids, fatty acids, cellular lipids, steroids, alcohols, eicosanoids, and long-chain fatty acids), immune and inflammatory response (such as complement and coagulation cascades, cytokine-cytokine receptor interaction, inflammatory mediator regulation of TRP channels, antigen processing and presentation, and T-cell receptor signaling pathway), liver fibrosis (such as ECM–receptor interactions), and cell death (PI3K-Akt signaling, apoptosis, and TGF-beta signaling pathways).

**FIGURE 8:**
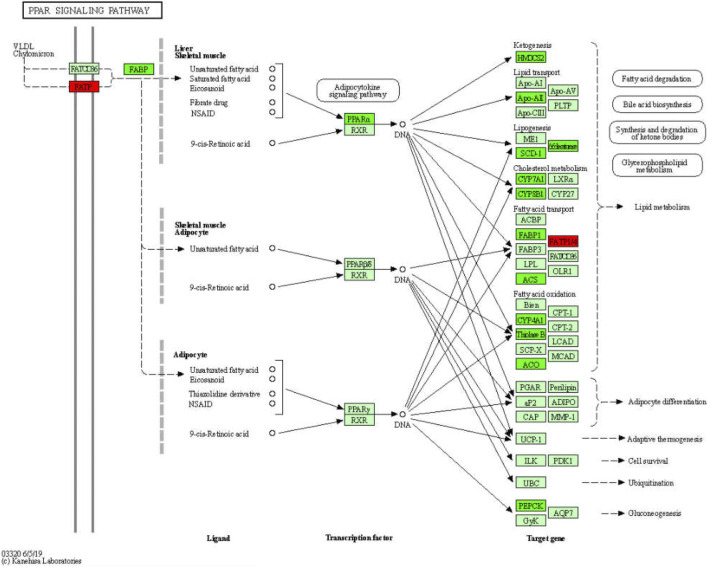
Significantly enriched KEGG pathways in PPAR signaling pathway.

The GO enrichment analysis revealed dozens of metabolic GO terms that were significantly enriched; these included organic acid metabolic process, carboxylic acid metabolic process, monocarboxylic acid metabolic process, lipid metabolic process, fatty acid metabolic process, cellular lipid metabolic process, steroid metabolic process, alcohol metabolic process, eicosanoid metabolic process, and long-chain fatty acid metabolic process. With KEGG pathway analysis, we also found dozens of metabolic pathways that were significantly enriched, including steroid hormone biosynthesis, metabolic pathways, retinol metabolism, arachidonic acid metabolism, glycine, serine, and threonine metabolism, primary bile acid biosynthesis, drug metabolism—other enzymes, metabolism of xenobiotics by cytochrome P450, linoleic acid metabolism, steroid biosynthesis, and starch and sucrose metabolism. Notably, the PPAR signaling pathway, which is involved in the metabolism of fatty acids, bile acids, lipids, glycerophospholipids, glucose, and other substances, was also significantly enriched (Ceni et al., 2014; Li et al., 2015; Liu et al., 2015; Pawlak et al., 2015). Specifically, we found that GDFMD could effectively reverse the abnormal expression of 20 genes in WD; among these the expression of 18 genes (*Apoa2*, *Acaa1b*, *Acsl1*, *Acox1*, *Ppara*, *Fads2*, *Scd2*, *Slc27a2*, *Pck1*, *Hmgcs2*, *Cyp7a1*, *Cyp4a31*, *Cyp4a14*, *Slc27a5*, *Slc27a1*, *Scd1*, *Cyp8b1*, and *Fabp1*) was low and that of 2 genes (*Cyp4a32* and *Cyp4a10*) was high in the Model group. A large body of evidence suggests that PPAR signaling pathway is involved in different liver diseases (Panebianco et al., 2017; Zhang et al., 2020), especially liver fibrosis (Chen et al., 2015; Pawlak et al., 2015; Chhimwal et al., 2020), as was also confirmed in the present study. Our results show that GDFMD can alleviate liver damage in WD by reversing the changes in the PPAR signaling pathway (Figure 8).

**FIGURE 9:**
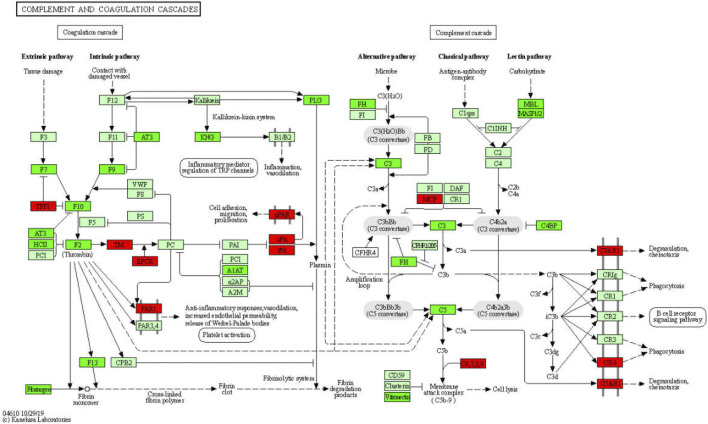
Significantly enriched KEGG pathways in complement and coagulation cascades.

GO enrichment analysis revealed that more than 327 DEGs are involved in the immune system process (*p* = 2.156e−14) and more than 120 DEGs are involved in the inflammatory response (*p* = 1.169e−12). It appears that both adaptive (*p* = 2.397e−06) and innate (*p* = 4.022e−06) immune responses are abnormal in WD. Several immune and inflammatory response pathways, such as complement and coagulation cascades, cytokine-cytokine receptor interaction, inflammatory mediator regulation of TRP channels, antigen processing and presentation, and T cell receptor signaling pathway, were enriched. We found that hundreds of immune factors, including 133 cytokines and cytokine receptors, 13 interleukins and interleukin receptors, 8 TNF family members and receptors, 10 TGF-β family members and receptors, and 34 chemokines and receptors, are dysregulated in WD, and that GDFMD could revert these dysregulations. Therefore, we hypothesize that WD can lead to the recruitment and induction of monocytes, T cells, eosinophils, and lymphocytes, among other immune cells. Previous studies have shown that the complement system is involved in a variety of chronic liver diseases (Bykov et al., 2007; Schmitt et al., 2012; Himoto et al., 2019). The activation of the complement and coagulation cascade leads to an inflammatory response, including degranulation, chemotaxis, and phagocytosis. Our study confirms these findings. We found that GDFMD inhibits the changes in the expression of *C3ar1*, *C4R*, and *C5ar1* caused by WD. We, therefore, speculate that liver injury in WD can be inhibited by GDFMD through the regulation of the immune and inflammatory response, particularly of the complement and coagulation cascades (Figure 9).

**FIGURE 10:**
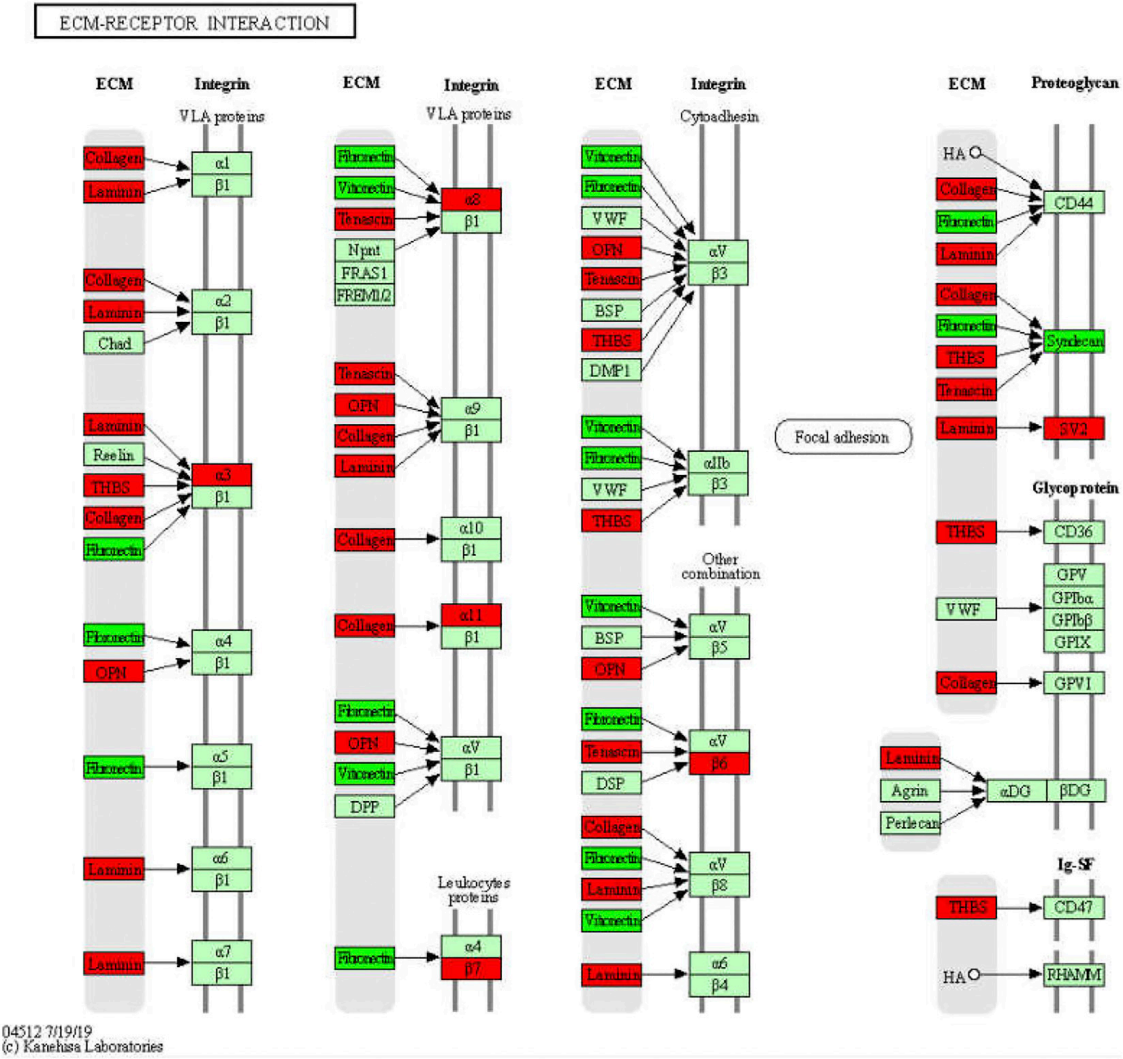
Significantly enriched KEGG pathways in ECM-receptors interaction.

During liver injury, the expression of and changes in various ECM components in fibrous tissues may convey important pathophysiological information, which is valuable for the development of new biomarkers and antifibrosis interventions. We identified that more than 31 ECM-receptors, except for SDC4, VTN, and FN1, were upregulated in WD (Figure 10). In particular, 11 collagens (*COL1A1, COL1A2, COL4A3, COL4A4, COL4A5, COL4A6, COL6A1, COL6A2, COL6A3, COL6A5, and COL9A3*)*, 5 integrins* (*ITGA11, ITGA3, ITGA8, ITGB6, and ITGB7*), 6 laminins (*LAMA2, LAMA4, LAMA5, LAMB1, LAMB2, and LAMC2*), and 3 thrombospondins (*THBS1, THBS2, and THBS3*) were upregulated in WD, and GDFMD could effectively reverse their abnormal expression levels. Collagen has been used as an important marker of liver fibrosis, and a large number of drugs have been developed based on the regulation of collagen, which can play an effective role in inhibiting liver fibrosis (Cho et al., 2000; Williams et al., 2001; Galli et al., 2002; Fontana et al., 2008). We also found that integrins α3, α8, α11, β6, and β7 were upregulated in WD. Popov and Sullivan demonstrated the role of β6 in liver fibrosis (Popov et al., 2008; Sullivan et al., 2010). In general, our results indicate that there is a systemic abnormal expression of ECM-receptors during the pathogenesis of WD. Reversal of these abnormalities could definitely be one of the mechanisms for the treatment of liver injury in WD.

Besides, we also identified many cell death-related genes and pathways that could be affected by GDFMD, such as the PI3K-Akt signaling pathway, apoptosis, and TGF-beta signaling pathway. Sixteen genes related to TGF-beta signaling pathway, namely *Ltbp1*, *Id3*, *Tgfb3*, *Thbs1*, *Dcn*, *Id4*, *Bambi*, *Tnf*, *Inhbc*, *Smad7*, *Bmp5*, *Inhbb*, *Gdf7*, *Bmp6*, *Id1*, and *Gdf6*, could be enriched, consistent with our findings in a previous study (Yang et al., 2018).

In conclusion, GDFMD may attenuate tx-j WD by altering metabolism, immune and tissue remodeling, and cell death-related pathways. This is a bioinformatics study to explore the unknown mechanism underlying the effect of a TCM. The results provide valuable information for further research and can provide a new model for development of novel drugs. Although most of our data are consistent with those reported in other studies, some limitations of this study are worth noting. First, the progression of WD may be related to age. In this study, DEGs were examined in adult male mice. Further investigation is needed to verify the candidate targets in different age groups. Second, in view of the regulation of protein translation and post-translational modification, we do not know the extent to which altered gene expression affects protein function. The functions of proteins encoded by these genes need to be further studied to provide new ideas for the treatment of WD.

## Conclusion

Through genetic engineering mouse model and RNA-seq, we preliminarily verified the efficacy and mechanism of GDFMD in the treatment of WD. The pathogenesis of WD and the treatment mechanism of GDFMD were interpreted from the whole genome level. However, further experimental is needed to verify for identified genes and pathways.

## Data Availability

The datasets presented in this study can be found in online repositories. The names of the repository/repositories and accession number(s) can be found below: https://bigd.big.ac.cn/bioproject/browse/PRJCA004081.

## References

[B1] Ashburner M. Ball C. A. Blake J. A. Botstein D. Butler H. Cherry J. M. (2000). Gene ontology: tool for the unification of biology. Nat. Genet. 25 (1), 25–29. 10.1038/75556 10802651 PMC3037419

[B2] Bader G. D. Hogue C. W. (2003). An automated method for finding molecular complexes in large protein interaction networks. BMC Bioinformatics 4, 2. 10.1186/1471-2105-4-2 12525261 PMC149346

[B3] Bandmann O. Weiss K. H. Kaler S. G. (2015). Wilson's disease and other neurological copper disorders. Lancet Neurol. 14 (1), 103–113. 10.1016/s1474-4422(14)70190-5 25496901 PMC4336199

[B4] Benjamini Y. Drai D. Elmer G. Kafkafi N. Golani I. (2001). Controlling the false discovery rate in behavior genetics research. Behav. Brain Res. 125 (1, 2), 279–284. 10.1016/s0166-4328(01)00297-2 11682119

[B5] Bykov I. Jauhiainen M. Olkkonen V. M. Saarikoski S. T. Ehnholm C. Junnikkala S. (2007). Hepatic gene expression and lipid parameters in complement C3−/− mice that do not develop ethanol-induced steatosis. J. Hepatol. 46 (5), 907–914. 10.1016/j.jhep.2006.11.020 17321001

[B6] Ceni E. Mello T. Galli A. (2014). Pathogenesis of alcoholic liver disease: role of oxidative metabolism. World J. Gastroenterol. 20 (47), 17756–17772. 10.3748/wjg.v20.i47.17756 25548474 PMC4273126

[B7] Chen L. Li L. Chen J. Li L. Zheng Z. Ren J. (2015). Oleoylethanolamide, an endogenous PPAR-α ligand, attenuates liver fibrosis targeting hepatic stellate cells. Oncotarget 6 (40), 42530–42540. 10.18632/oncotarget.6466 26729705 PMC4767450

[B8] Chen Q. Wu F. Wang M. Dong S. Liu Y. Lu Y. (2016). Transcriptional profiling and miRNA-target network analysis identify potential biomarkers for efficacy evaluation of Fuzheng-Huayu Formula-treated hepatitis B caused liver cirrhosis. Int. J. Mol. Sci. 17 (6), 883. 10.3390/ijms17060883 PMC4926417 27271613

[B9] Chen S. Zhou Y. Chen Y. Gu J. (2018). fastp: an ultra-fast all-in-one FASTQ preprocessor. Bioinformatics 34 (17), i884–i890. 10.1093/bioinformatics/bty560 30423086 PMC6129281

[B10] Chhimwal J. Sharma S. Kulurkar P. Patial V. (2020). Crocin attenuates CCl4-induced liver fibrosis via PPAR-γ mediated modulation of inflammation and fibrogenesis in rats. Hum. Exp. Toxicol. 39 (12), 1639–1649. 10.1177/0960327120937048 32633567

[B11] Cho J.-J. Hocher B. Herbst H. Jia J.-D. Ruehl M. Hahn E. G. (2000). An oral endothelin-A receptor antagonist blocks collagen synthesis and deposition in advanced rat liver fibrosis. Gastroenterology 118 (6), 1169–1178. 10.1016/s0016-5085(00)70370-2 10833492

[B12] European Association for Study of Liver (2012). EASL clinical practice Guidelines: wilson's disease. J. Hepatol. 56 (3), 671–685. 10.1016/j.jhep.2011.11.007 22340672

[B13] Ferenci P. Stremmel W. Członkowska A. Szalay F. Viveiros A. Stättermayer A. F. (2019). Age and sex but not ATP7B genotype effectively influence the clinical phenotype of Wilson disease. Hepatology 69 (4), 1464–1476. 10.1002/hep.30280 30232804

[B14] Fontana R. J. Goodman Z. D. Dienstag J. L. Bonkovsky H. L. Naishadham D. Sterling R. K. (2008). Relationship of serum fibrosis markers with liver fibrosis stage and collagen content in patients with advanced chronic hepatitis C. Hepatology 47 (3), 789–798. 10.1002/hep.22099 18175357

[B15] Galli A. Crabb D. W. Ceni E. Salzano R. Mello T. Svegliati–Baroni G. (2002). Antidiabetic thiazolidinediones inhibit collagen synthesis and hepatic stellate cell activation *in vivo* and *in vitro* . Gastroenterology 122 (7), 1924–1940. 10.1053/gast.2002.33666 12055599

[B16] Gerosa C. Fanni D. Congiu T. Piras M. Cau F. Moi M. (2019). Liver pathology in Wilson's disease: from copper overload to cirrhosis. J. Inorg. Biochem. 193, 106–111. 10.1016/j.jinorgbio.2019.01.008 30703747

[B17] Himoto T. Hirakawa E. Fujita K. Sakamoto T. Nomura T. Morishita A. (2019). Complement component 3 as a surrogate hallmark for metabolic abnormalities in patients with chronic hepatitis C. Ann. Clin. Lab. Sci. 49 (1), 79–88. 30814081

[B18] Kanehisa M. Goto S. (2000). KEGG: kyoto encyclopedia of genes and genomes. Nucl. Acids Res. 28 (1), 27–30. 10.1093/nar/28.1.27 10592173 PMC102409

[B19] Kim D. Langmead B. Salzberg S. L. (2015). HISAT: a fast spliced aligner with low memory requirements. Nat. Methods 12 (4), 357–360. 10.1038/nmeth.3317 25751142 PMC4655817

[B20] Li H. Wang T. Xu C. Wang D. Ren J. Li Y. (2015). Transcriptome profile of liver at different physiological stages reveals potential mode for lipid metabolism in laying hens. BMC Genomics 16, 763. 10.1186/s12864-015-1943-0 26452545 PMC4600267

[B21] Liu Z.-M. Hu M. Chan P. Tomlinson B. (2015). Early investigational drugs targeting PPAR-α for the treatment of metabolic disease. Expert Opin. Investig. Drugs 24 (5), 611–621. 10.1517/13543784.2015.1006359 25604802

[B22] Livak K. J. Schmittgen T. D. (2001). Analysis of relative gene expression data using real-time quantitative PCR and the 2^−ΔΔCT^ method. Methods 25 (4), 402–408. 10.1006/meth.2001.1262 11846609

[B23] Mortazavi A. Williams B. A. McCue K. Schaeffer L. Wold B. (2008). Mapping and quantifying mammalian transcriptomes by RNA-Seq. Nat. Methods 5 (7), 621–628. 10.1038/nmeth.1226 18516045 PMC13303166

[B24] Nikolayeva O. Robinson M. D. (2014). edgeR for differential RNA-seq and ChIP-seq analysis: an application to stem cell biology. Methods Mol. Biol. 1150, 45–79. 10.1007/978-1-4939-0512-6_3 24743990

[B25] Panebianco C. Oben J. A. Vinciguerra M. Pazienza V. (2017). Senescence in hepatic stellate cells as a mechanism of liver fibrosis reversal: a putative synergy between retinoic acid and PPAR-gamma signalings. Clin. Exp. Med. 17 (3), 269–280. 10.1007/s10238-016-0438-x 27655446

[B26] Pawlak M. Lefebvre P. Staels B. (2015). Molecular mechanism of PPARα action and its impact on lipid metabolism, inflammation and fibrosis in non-alcoholic fatty liver disease. J. Hepatol. 62 (3), 720–733. 10.1016/j.jhep.2014.10.039 25450203

[B27] Pertea M. Kim D. Pertea G. M. Leek J. T. Salzberg S. L. (2016). Transcript-level expression analysis of RNA-seq experiments with HISAT, StringTie and Ballgown. Nat. Protoc. 11 (9), 1650–1667. 10.1038/nprot.2016.095 27560171 PMC5032908

[B28] Pertea M. Pertea G. M. Antonescu C. M. Chang T.-C. Mendell J. T. Salzberg S. L. (2015). StringTie enables improved reconstruction of a transcriptome from RNA-seq reads. Nat. Biotechnol. 33 (3), 290–295. 10.1038/nbt.3122 25690850 PMC4643835

[B29] Popov Y. Patsenker E. Stickel F. Zaks J. Bhaskar K. R. Niedobitek G. (2008). Integrin αvβ6 is a marker of the progression of biliary and portal liver fibrosis and a novel target for antifibrotic therapies. J. Hepatol. 48 (3), 453–464. 10.1016/j.jhep.2007.11.021 18221819

[B30] Reed E. Lutsenko S. Bandmann O. (2018). Animal models of Wilson disease. J. Neurochem. 146 (4), 356–373. 10.1111/jnc.14323 29473169 PMC6107386

[B31] Robinson M. D. McCarthy D. J. Smyth G. K. (2010). edgeR: a Bioconductor package for differential expression analysis of digital gene expression data. Bioinformatics 26 (1), 139–140. 10.1093/bioinformatics/btp616 19910308 PMC2796818

[B32] Schmitt J. Roderfeld M. Sabrane K. Zhang P. Tian Y. Mertens J. C. (2012). Complement factor C5 deficiency significantly delays the progression of biliary fibrosis in bile duct-ligated mice. Biochem. Biophys. Res. Commun. 418 (3), 445–450. 10.1016/j.bbrc.2012.01.036 22277671

[B33] Shannon P. Markiel A. Ozier O. Baliga N. S. Wang J. T. Ramage D. (2003). Cytoscape: a software environment for integrated models of biomolecular interaction networks. Genome Res. 13 (11), 2498–2504. 10.1101/gr.1239303 14597658 PMC403769

[B34] Sullivan B. P. Weinreb P. H. Violette S. M. Luyendyk J. P. (2010). The coagulation system contributes to αVβ6 integrin expression and liver fibrosis induced by cholestasis. Am. J. Pathol. 177 (6), 2837–2849. 10.2353/ajpath.2010.100425 21037076 PMC2993265

[B35] Szklarczyk D. Morris J. H. Cook H. Kuhn M. Wyder S. Simonovic M. (2017). The STRING database in 2017: quality-controlled protein-protein association networks, made broadly accessible. Nucl. Acids Res. 45 (D1), D362–D368. 10.1093/nar/gkw937 27924014 PMC5210637

[B36] Tang L. Liu D. Li R. Dong T. Chen Y. Jiang H. (2018). The protective effect and mechanism of Gandou Fumu Decoction on liver fibrosis in TX mice. Chin. J. Integrated Tradition. Chin. West. Med. 38, 1461–1466. 10.7661/j.cjim.20181023.312

[B37] Tang L. Yang W. Xie W. Tao C. Dong T. Chen Y. (2019). Effects of Gandou Fumu Decoction on the expression of TβRI, TβRII and Smad4 in liver tissues of TX mice with Wilson’s disease liver fibrosis. J. Zhonghua Med. 34 (09), 4043–4047.

[B38] Williams E. J. Benyon R. C. Trim N. Hadwin R. Grove B. H. Arthur M. J. (2001). Relaxin inhibits effective collagen deposition by cultured hepatic stellate cells and decreases rat liver fibrosis *in vivo* . Gut 49 (4), 577–583. 10.1136/gut.49.4.577 11559657 PMC1728476

[B39] Yang W. Fang F. Wang M. Wang H. Dong T. (2014). Clinical study of Gandou Fumu Decoction in the treatment of Wilson's disease liver fibrosis. Clin. J. Tradition. Chin. Med. 26, 1111–1113. 10.16448/j.cjtcm.2014.11.010

[B40] Yang W. Tang L. Xie W. Liu D. Dong T. (2018). Effect of Gandou Fumu Decoction on TGF-β_1/Smad signaling pathway in TX mice with liver fibrosis. J. Integrated Traditional Chin. West. Med. Cardio-Cerebrovasc. Dis. 16, 404–407. 10.3969/j.issn.1672-1349.2018.04.006

[B41] Yu G. Wang L.-G. Han Y. He Q.-Y. (2012). clusterProfiler: an R package for comparing biological themes among gene clusters. OMICS: A J. Integr. Biol. 16, 284–287. 10.1089/omi.2011.0118 PMC3339379 22455463

[B42] Zhang J. Du H. Shen M. Zhao Z. Ye X. (2020). Kangtaizhi granule alleviated nonalcoholic fatty liver disease in high-fat diet-fed rats and HepG2 cells via AMPK/mTOR signaling pathway. J. Immunol. Res. 2020, 3413186. 10.1155/2020/3413186 32884949 PMC7455821

