# Supplementary material for: Comprehensive RNA-Seq Analysis of Potential Therapeutic Targets of Gan–Dou–Fu–Mu Decoction for Treatment of Wilson Disease Using a Toxic Milk Mouse Model
Source: Front Pharmacol. 2021 Apr 15;12:622268. doi: 10.3389/fphar.2021.622268 (PMC8082393; doi:10.3389/fphar.2021.622268)
Supplement: Supplementary file 1 [file image1.pdf]

Figure S1 The MRM diagram of the seven components in mixed reference solution and GDFMD.

(A: Gallic acid, B: Paeoniflorin, C: Resveratrol, D: Quercetin, E: Notoginsenoside, F: Saikosaponin A, G: Curcumin. 1: Mixed reference solution, 2: GDFMD sample solution).

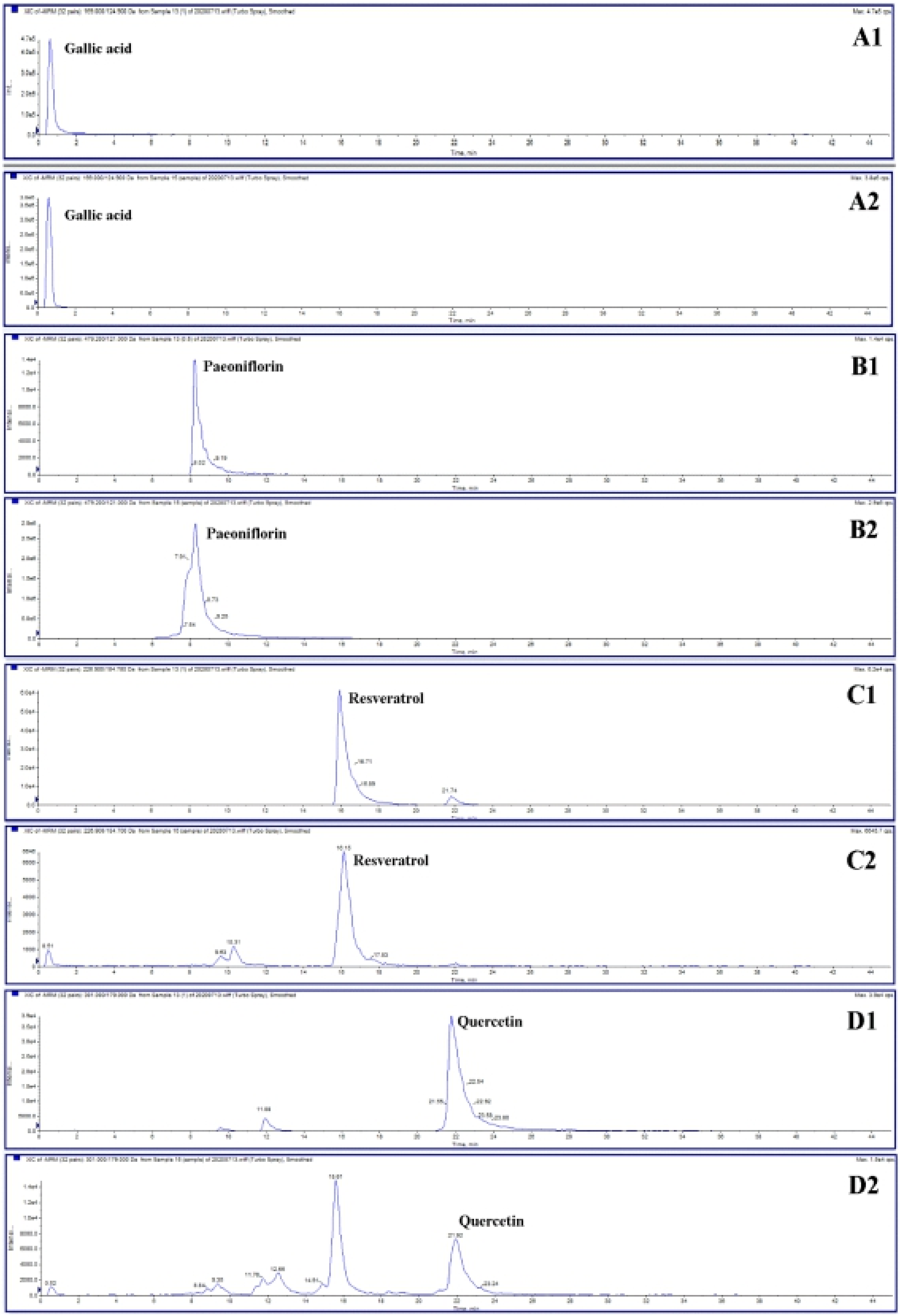

Continue

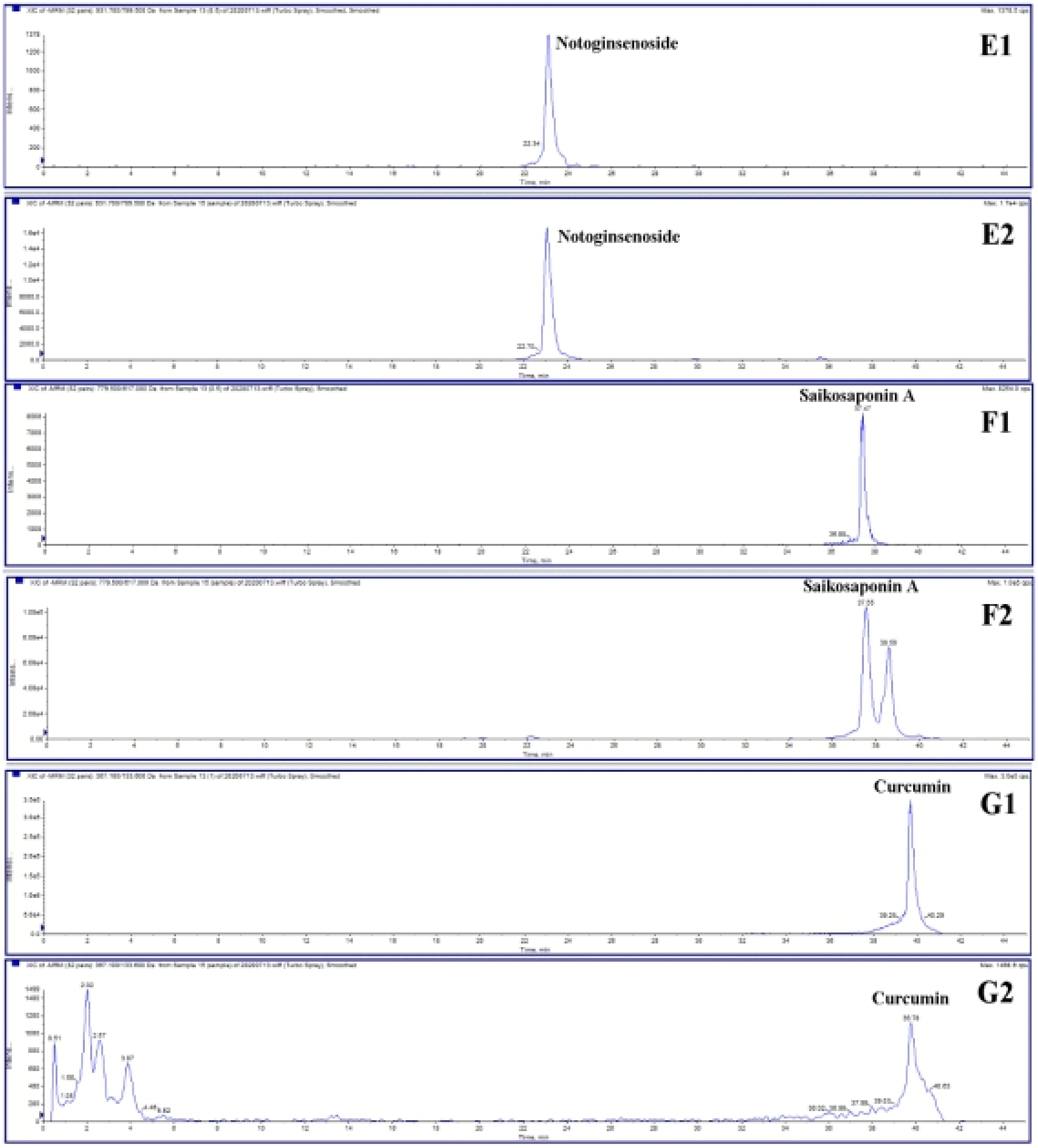
Notoginsenoside
E1
Notoginsenoside
E2
Saikosaponin A
F1
Saikosaponin A
F2
Curcumin
G1
Curcumin
G2
